# Whole-Genome Sequence of the Clinical Strain *Corynebacterium argentoratense* DSM 44202, Isolated from a Human Throat Specimen

**DOI:** 10.1128/genomeA.00793-13

**Published:** 2013-10-03

**Authors:** Christina Bomholt, Alina Glaub, Kerstin Gravermann, Andreas Albersmeier, Karina Brinkrolf, Christian Rückert, Andreas Tauch

**Affiliations:** Institut für Genomforschung und Systembiologie, Centrum für Biotechnologie, Universität Bielefeld, Bielefeld, Germany

## Abstract

*Corynebacterium argentoratense* is part of the human skin microbiota and is occasionally detected in the upper respiratory tract of patients suffering from tonsillitis. The complete DNA sequence of the type strain DSM 44202 comprises 2,031,902 bp, yielding the smallest genome sequenced thus far for a corynebacterium associated with humans.

## GENOME ANNOUNCEMENT

*Corynebacterium argentoratense* was first identified in clinical specimens of inpatients at the University Hospital of Strasbourg, France (1, 2). Four isolates were obtained from patients suffering from tonsillitis, and by standard chemotaxonomic studies these isolates were subsequently characterized as members of a new species within the genus *Corynebacterium* (2). A close relative in the phylogenetic tree is *Corynebacterium kutscheri*, which is commonly isolated from the oral cavities of mice and rats (3). Clinical *C. argentoratense* strains were mainly found in the upper respiratory tract of humans (2, 4) but were also found in a human blood culture (5) and as part of a mucosal biofilm in adenoid tissues of children with otitis media (6). Furthermore, operational taxonomic units showing 99% nucleotide sequence identity to the 16S rRNA gene of *C. argentoratense* were obtained from several distinct skin sites of healthy humans in the course of the Human Microbiome Project (7) and in a study of the skin microbiome associated with atopic dermatitis in children (8). To get detailed insights into the gene repertoire of this rarely detected pathogen, we sequenced the genome of the type strain of *C. argentoratense*, which was initially named IBS B10697 (2).

*C. argentoratense* strain DSM 44202 (IBS B10697, CIP 104296, ATCC 51927) was obtained as a lyophilized culture from the Leibniz Institute DSMZ (Braunschweig). The bacterium was grown in brain yeast (BY) medium containing 37 g/liter brain heart broth and 10 g/liter yeast extract. Genomic DNA was purified with the Genomic-tip 500/G system and the Genomic DNA buffer set according to the protocols of the *Qiagen Genomic DNA Handbook* (http://www.qiagen.com/Knowledge-and-Support/Resource-Center/Resource-Search/?q=Genomic+DNA+handbook%3b&l=en%3b). Sequencing libraries were generated by following the workflow of the Nextera DNA sample preparation kit (Illumina). The constructed genomic library was sequenced by the paired-end approach (2 × 250) using the MiSeq reagent kit v2 and the MiSeq benchtop sequencer (Illumina), resulting in 1,188,280 reads and 272,951,470 detected bases. The reads were assembled with Roche’s GS De Novo Assembler (version 2.8) to yield 20 contigs in five scaffolds. Remaining gaps in the genome sequence were closed *in silico* with the Consed assembly software package (version 24) (9). The deduced chromosome of *C. argentoratense* DSM 44202 has a size of 2,031,902 bp with an average G+C content of 58.9%. A regional and functional annotation was added to the genome sequence by the NCBI Prokaryotic Genome Annotation Pipeline using the GeneMarkS+ software (version 2.1), thereby revealing 1,875 protein-coding regions, 2 pseudogenes, 50 tRNAs, and 4 rRNA operons. In conclusion, *C. argentoratense* DSM 44202 harbors the smallest completely sequenced chromosome of corynebacterial species, which have been genetically characterized so far as having genome sizes ranging from 2,279,118 bp (*Corynebacterium pseudotuberculosis* 1/06-A) (10) to 3,433,007 bp (*Corynebacterium variabile* DSM 44702) (11). Moreover, the genome of *C. argentoratense* DSM 44202 lacks typical corynebacterial antibiotic resistance genes (12), supporting previous *in vitro* data that demonstrated a broad antimicrobial susceptibility of clinical *C. argentoratense* isolates (4).

### Nucleotide sequence accession number.

This genome project has been deposited in GenBank under the accession number CP006365.
